# Effect of Climate Change on Invasion Risk of Giant African Snail (*Achatina fulica* Férussac, 1821: Achatinidae) in India

**DOI:** 10.1371/journal.pone.0143724

**Published:** 2015-11-30

**Authors:** Roshmi Rekha Sarma, Madhushree Munsi, Aravind Neelavara Ananthram

**Affiliations:** 1 Suri Sehgal Center for Biodiversity and Conservation, Ashoka Trust for Research in Ecology and the Environment (ATREE), Royal Enclave, Srirampura, Jakkur PO, Bangalore, 560064, India; 2 Manipal University, Manipal, 576104, Karnataka, India; Australian Museum, AUSTRALIA

## Abstract

The Giant African Snail (*Achatina fulica*) is considered to be one the world’s 100 worst invasive alien species. The snail has an impact on native biodiversity, and on agricultural and horticultural crops. In India, it is known to feed on more than fifty species of native plants and agricultural crops and also outcompetes the native snails. It was introduced into India in 1847 and since then it has spread all across the country. In this paper, we use ecological niche modeling (ENM) to assess the distribution pattern of Giant African Snail (GAS) under different climate change scenarios. The niche modeling results indicate that under the current climate scenario, Eastern India, peninsular India and the Andaman and Nicobar Islands are at high risk of invasion. The three different future climate scenarios show that there is no significant change in the geographical distribution of invasion prone areas. However, certain currently invaded areas will be more prone to invasion in the future. These regions include parts of Bihar, Southern Karnataka, parts of Gujarat and Assam. The Andaman and Nicobar and Lakshadweep Islands are highly vulnerable to invasion under changed climate. The Central Indian region is at low risk due to high temperature and low rainfall. An understanding of the invasion pattern can help in better management of this invasive species and also in formulating policies for its control.

## Introduction

One of the greatest threats to global biodiversity, agriculture, livelihoods, human and animal health, and forestry is invasive alien species [1–3]. Invasive alien species affect not only the environment or ecology, but also the local economy [3]. Ever-growing international trade, increased transportation and deliberate introductions have facilitated invasions at an unprecedented rate across the globe [4,5]. Another factor that has great implications for biodiversity, environment and human health is climate change. Invasive alien species acting synergistically with climate change might be expected to have much larger impact on local ecosystem than either acting alone [6]. It has been hypothesized that climate change will aggravate the impacts of “non-native/exotic/alien” species naturalization and subsequent invasion across communities and ecosystems in the new ranges, thus threatening native biodiversity [7–15] and human well-being [16]. Identifying the probable future distribution of invasive alien species is of paramount importance for early detection, prioritization of regions for conservation and effective management of invasive species [17,18].

The giant African snail (*Achatina fulica* Férussac, 1821: Achatinidae) is a highly invasive terrestrial snail native to East Africa. According to the global invasive species database, *A*. *fulica* is the second worst invasive alien species in the world. It has now invaded most parts of the world with particular impact in tropical and subtropical regions [19]. It was introduced either accidentally along with agriculture or horticulture produce or deliberately as pets, or ornamental, medicinal or food resources [20]. Given the very high density of this large pest, it is likely to have a great impact on the native biodiversity and ecosystems [20]. *A*. *fulica* has been recognized as a major agricultural and garden pest [21,22]. It is known to consume more than 50 species of native plants, agricultural and horticultural crops, modify habitats and outcompete native snails [22,23]. The species reproduces rapidly under optimal field conditions and can reach high densities and biomass in a very short time [22,24,25]. *A*. *fulica* spreads the spores of pathogens of a variety of cultivated plants in the introduced range [22,26–29]. It is also a vector of the rat lungworm, *Angiostrongylus cantonensis*, which causes eosinophilic meningoencephalitis in humans [30,31]. Apart from economic loss and human health issues caused by *A*. *fulica*, they are also a general nuisance to people. According to Mead [26], there are reports of car accidents due to cars skidding on crushed snails on roads.

The pioneering British malacologist, William Henry Benson, brought a pair of *A*. *fulica* to India from Mauritius, and handed them to a friend and neighbor before leaving the country. It was this friend who released the snails in his garden in Kolkata, eastern India [32]. Later, in 1858, Benson reported that *A*. *fulica* had become well established in Kolkata [33,34]. *A*. *fulica* has been considered as a serious pest to small-scale agriculture, horticulture and home gardens [22]. In India, the spread of *A*. *fulica* was primarily mediated by humans. For example, a farmer has introduced it from Orissa to his farm in Araku valley in Andhra Pradesh in 1996 [35]; a blacksmith was believed to have brought *A*. *fulica* as a pet from Kolkata to north Bihar some time during 60s [36]. Since then it has invaded many parts of both the states. This could be the case for many other sites. The snail being large in size and attractive might have fascinated pet keepers and hence got transported to many parts of the country from Kolkata. By mid-20^th^ century, *A*. *fulica* attained serious pest status in India and caused severe damage to agricultural crops [37–39]. Many studies in India have reported the impact of *A*. *fulica* invasion on horticultural and agricultural crops; however, none have quantified the exact economic loss [40]. In the late 1970s, this snail was reported to damage ornamental and vegetable crops in Bangalore [38]. Since then, several reports have shown that *A*. *fulica* causes serious damage to variety of horticultural and agricultural crops viz., mulberry [41], betel-vine, capsicum, areca, banana, tomato [42], vanilla [39] and vegetable crops such as potato, spinach, radish and tomato [43]. Raut & Ghose [25] stated that nearly 90% plants cultivated in India were eaten by *A*. *fulica*. The pest generally feeds on the succulent parts of the plants and damages the small and tender parts and seedlings completely. They feed not only on leaves but also on flowers, buds, tender shoots and fruits. Given the broad spectrum of food choice and increased precipitation in Southern India due to climate change, the invasion of *A*. *fulica* might exacerbate the situation. At present, the range has extended through much of India, Nepal, Bhutan, Bangladesh and Sri Lanka [44]. A recent genetic study showed that all *A*. *fulica* now occurring throughout South Asia, Southeast Asia and the Pacific Region are derived from a single haplotype [45].

In this paper, we assess the potential distribution of *A*. *fulica* under present and future climate change scenarios using ecological niche modeling tools. The ecological niche models (ENM) integrate species occurrence records with climatic and other environmental variables and generate maps of species’ potential distribution [46]. Of late, the ENM approach is being increasingly used to map potential distributions of invasive species under present and future climate change scenarios [47–51]. In this study, we used maximum entropy modeling (MaxEnt) to predict the invasion potential of the introduced invasive snail species, *A*. *fulica* in India and develop a risk map of areas likely to be invaded in the future, which can be used for prioritization and effective management of this species. The specific objectives of this paper were (1) to generate a map of the potential present distribution of *A*. *fulica* in India, (2) to assess the impact of climate change on the potential invasion patterns and (3) to identify bioclimatic factors that contribute to invasion of *A*. *fulica*.

## Materials and Methods

### *Achatina fulica* distribution data

A total of 231 records have been collated into the Achatina database. These came from two sources, a) for the global distribution the data was downloaded from GBIF, and b) for India, the sources were: i) published records on giant African snails in journals, books and reports, ii) specimens observed during field survey (mostly opportunistic sampling) by the authors, and iii) from the Zoological Survey of India Museum. Distribution data collected from these sources was converted into an MS excel format with specimen location, latitude, longitude, altitude and data source. All data were geocoded using Google Earth and/or Survey of India toposheets. The Garmin GPS was used to record the site of occurrence of each specimen for the primary data. The data for *A*. *fulica* distribution India can be accessed at: http://indiabiodiversity.org/map?layers=lyr_407_achatina_fulica&title=Distribution

### Environmental data

To develop the niche model, present environmental data and future predictions were downloaded from the Worldclim database website. For present (1950–2000) environmental data, a total of 19 bioclimatic layers were downloaded from Worldclim database version 1.4 [52] (http://www.worldclim.org/; Table 1). We used Intergovernmental Panel on Climate Change (IPCC) data in its fifth Assessment Report (AR5) [53] for three different future climate scenarios. These were Representative Concentration Pathway (RCP) scenarios (RCP 4.5, RCP 6.0, RCP 8.5)—each of which is based on one of three different levels of radiative forcing. These RCPs were developed by three different institutes. RCP 8.5 is characterized by increased greenhouse gas emissions throughout the 21st century that lead to high greenhouse gas concentrations over time. This RCP assumes there are no policy changes to reduce emissions in the future [54]. RCP 6.0 is Intermediate emission scenario where total radiative forcing is stabilized shortly after year 2100 by using range of technologies and strategies for reducing greenhouse gas emissions in the future. RCP 4.5 is a more optimistic scenario with emissions peak around 2040 and then decline [54]. All data used for ENM had spatial resolution of 1km^2^ (30 arc seconds).

**Table 1:** Environmental Data used for ENM for *A*. *fulica*. The highlighted variables were included in the model after testing for correlation between variables.

Codes	Variables
**BIO1**	**Annual Mean Temperature**
BIO2	Mean Diurnal Range (Mean of monthly (max temp—min temp))
BIO3	Isothermality (BIO2/BIO7) (*100)
**BIO4**	Temperature Seasonality (standard deviation *100)
BIO5	Max Temperature of Warmest Month
BIO6	Min Temperature of Coldest Month
**BIO7**	**Temperature Annual Range (BIO5-BIO6)**
BIO8	Mean Temperature of Wettest Quarter
BIO9	Mean Temperature of Driest Quarter
BIO10	Mean Temperature of Warmest Quarter
BIO11	Mean Temperature of Coldest Quarter
**BIO12**	**Annual Precipitation**
BIO13	Precipitation of Wettest Month
**BIO14**	**Precipitation of Driest Month**
**BIO15**	**Precipitation Seasonality (Coefficient of Variation)**
BIO16	Precipitation of Wettest Quarter
BIO17	Precipitation of Driest Quarter
BIO18	Precipitation of Warmest Quarter
**BIO19**	**Precipitation of Coldest Quarter**

### Ecological Niche Modeling

Maximum entropy modeling was used with the MaxEnt algorithm (version 3.3.3k) [55] for quantifying relative risk of invasion and mapping the potential geographic distribution of *A*. *fulica* in India. The selection of MaxEnt is based on following reasons: (1) it is a presence-only modeling algorithm (i.e. absence data are not required); (2) the performance is relatively better than other modeling methods [56]; and (3) the model is hardly influenced by small sample sizes and hence prediction will be relatively robust [50,57]. MaxEnt estimates the probability of presence of a species based on presence records and randomly generated background points by finding the maximum entropy distribution [55]. MaxEnt uses a regularization parameter to control over-fitting and can handle both categorical and continuous variables [55]. It uses five different features viz., linear, quadratic, product, threshold, and hinge, to constrain the geographical distribution of a species. We have used “logistic” output format in MaxEnt, which is a continuous map with an estimated probability of species’ presence between 0 and 1, which allows us to make distinctions between the suitability of different areas [58]. All 19 bioclim layers were tested for collinearity by examining pairwise correlations between them. When a pair of variables had a Pearson’s correlation coefficient >0.75, one of the two variables was removed and finally only seven variables were used for ENM.

A jackknife procedure was used to calculate the significance of the contribution of each variable to the model. The area under the Receiver Operating Characteristic curve (abbreviated as AUC) [59] was used to evaluate model performance. The AUC is a threshold-independent measure of a model’s ability to discriminate presence from absence (or background). It varies from 0.5 to 1.0; an AUC value of 0.5 shows that model predictions are not better than random, values <0.5 are worse than random, 0.5–0.7 signifies poor performance, 0.7–0.9 signifies reasonable/ moderate performance and >0.9 indicates high model performance [60]. Model validation was performed using the ‘sub-sampling’ procedure in MaxEnt. 75% of the *A*. *fulica* data were used for model calibration and the remaining 25% for model validation. Ten replicates were run and average AUC values for training and test datasets were calculated. Maximum iterations were set at 5000. Percent variable contribution and jackknife procedures in MaxEnt were used to investigate the relative importance of different bioclimatic predictors. For the present study, we selected five arbitrary categories of the risk of invasion by *A*. *fulica*: very low (0.10–0.25), low (0.25–0.50), moderate (0.50–0.70), high (0.70–0.90) and very high (>0.90) based on predicted habitat suitability as per Kumar et al. [61]. All GIS analysis was conducted in ArcGIS and statistical analysis was done in PAST 3.

## Results

### Current invasion pattern

**Fig 1:**
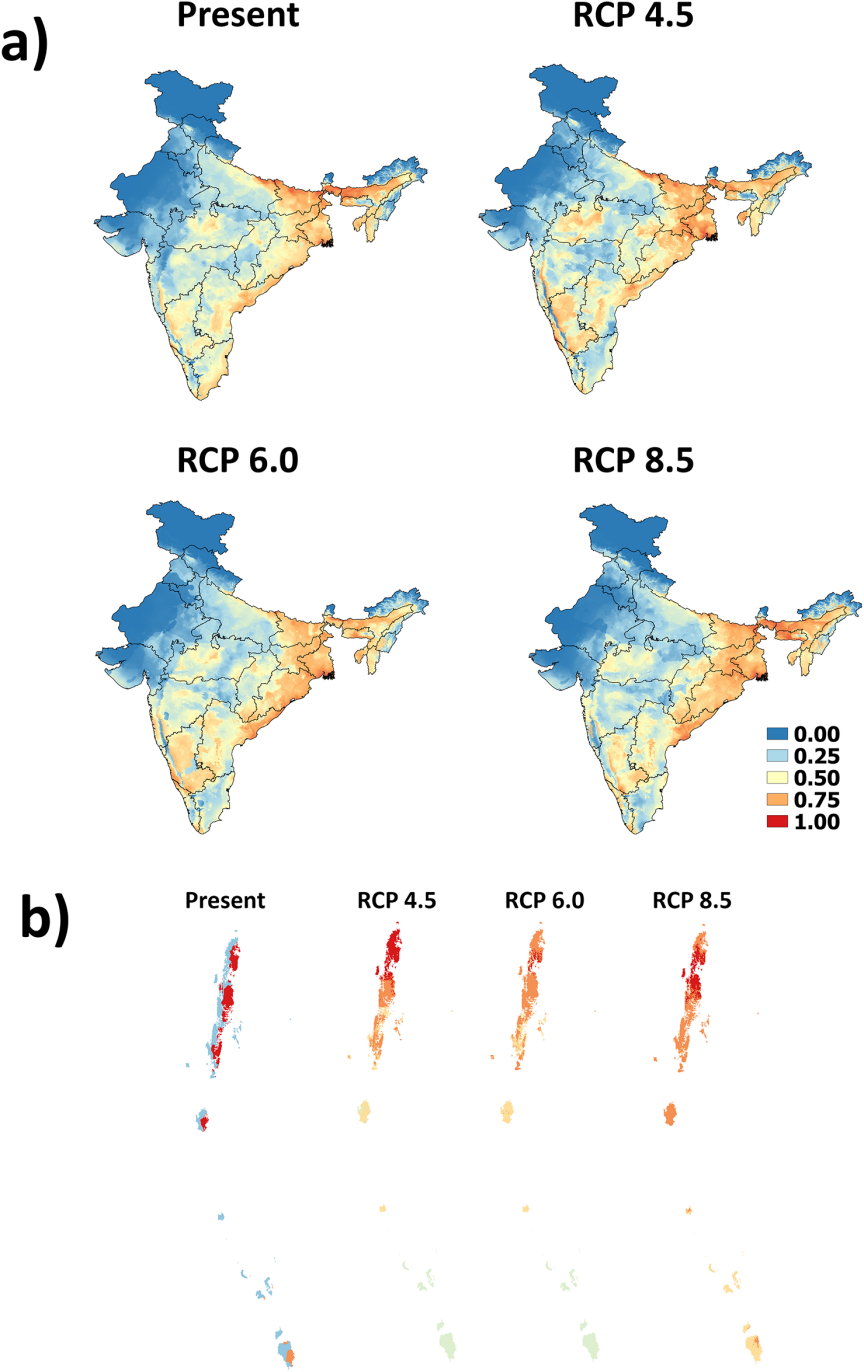
Potential distribution of *A*.*fulica* under current, and three climate change scenario (RCP 4.5, 6.0 and 8.0) a) for mainland India and b) Andaman and Nicobar Islands. Legend: Blue to red colour indicates unsuitable to highly suitable areas

**Fig 2:**
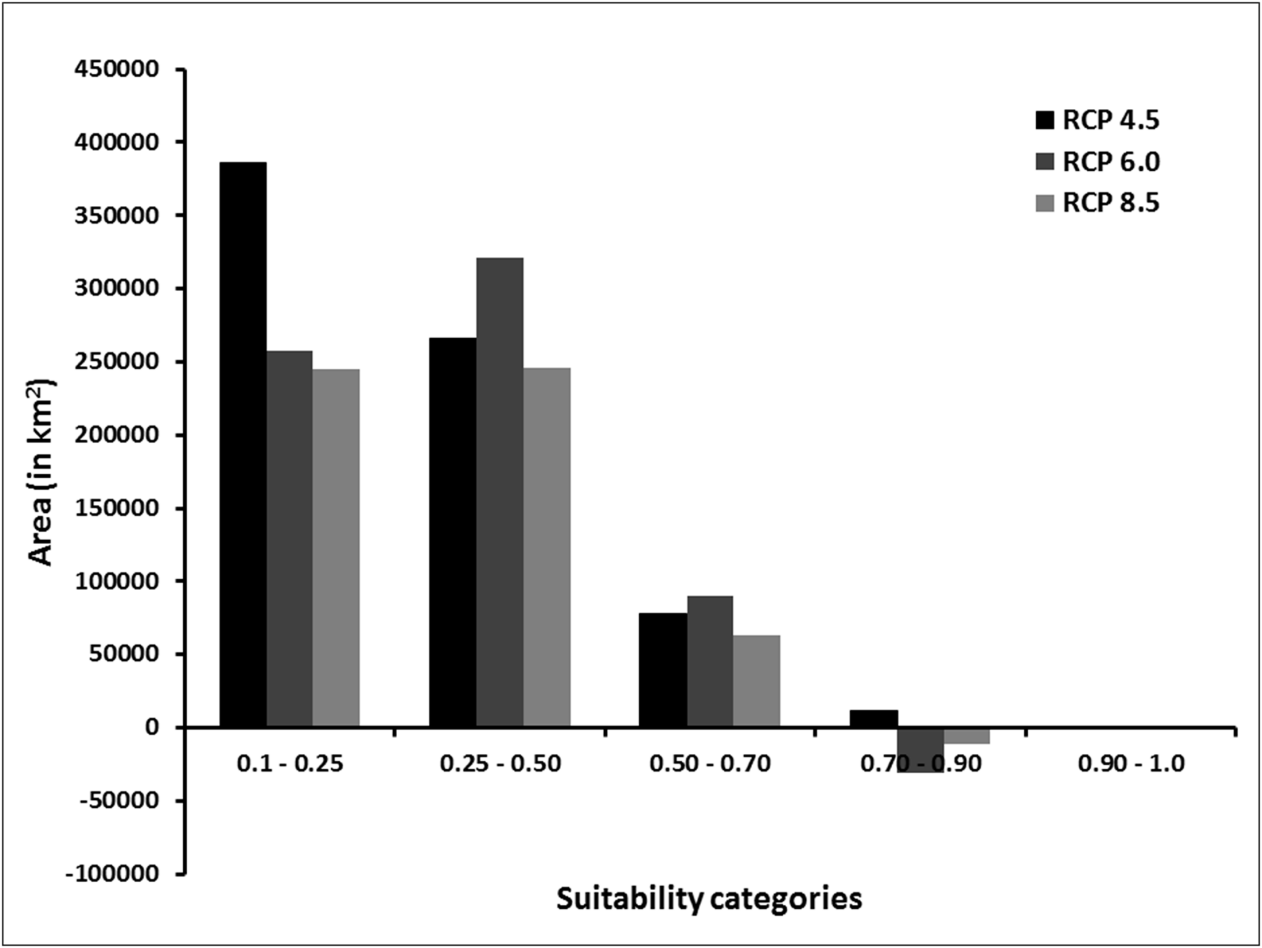
Percent difference in the area of different suitability category between RCP 4.5, RCP and 6.0 and RCP 8.0 and current scenario for *A*.*fulica*.

The MaxEnt model for *A*. *fulica* predicts three potential hotspots for invasion viz., western India, parts of peninsular India and eastern India. In western India, the Coast, especially in Kerala and parts of Karnataka, are most susceptible to invasion. In peninsular India, parts of southern and northwestern Karnataka, parts of eastern Tamil Nadu and coastal Andhra Pradesh have high risk of invasion under current climate scenario. In eastern India, the Tarai region, the Gangetic region of Bihar, West Bengal and Uttar Pradesh, the Brahmaputra river basin (in Assam), Meghalaya and Tripura have high (0.50–0.70 suitability scores) to very high (>0.70 suitability scores) probability of invasion. The low to moderate risk region extends till Odisha, parts of Andhra Pradesh in the East Coast and the Eastern Ghats (Fig 1a). The Andaman and Nicobar and Lakshadweep Islands also have very high risk of invasion (Fig 1b). The total area at risk of invasion is 2,003,265 km^2^ (Fig 2), of which 362,553 km^2^ (4.24%) has moderate risk, 112,759 km^2^ (1.32%) has high-risk and 98 km^2^ (<1%) has very high risk (Table 2).

**Table 2:** Area with suitability scores under different climate scenarios. The percentage is given in the parenthesis. The value less the 0.10 is not given.

Range	Present	RCP 4.5	RCP 6.0	RCP 8.5	RCP 4.5-Current	RCP 6.0-Current	RCP 8.5-Current
**Very Low (0.10—0.25)**	1,005,583 (11.76%)	1,391,342 (15.64%)	1,263,375 (14.20%)	1250,113 (14.05%)	385,759 (3.88%)	257,792 (2.44%)	244,530 (2.29%)
**Low (0.25—0.50)**	522,272 (6.11%)	788,082 (8.86%)	843,751 (9.48%)	768,520 (8.64%)	265,810 (2.75%)	321,479 (3.38%)	246,248 (2.53%)
**Moderate (0.50—0.70)**	362,553 (4.24%)	440,610 (4.95%)	452,319 (5.08%)	425,832 (4.79%)	78,057 (0.71%)	89,766 (0.84%7)	63,279 (0.55%)
**High (0.70—0.90)**	112,759 (1.32%)	124,382 (1.40%)	81,714 (0.92%)	101,299 (1.14%)	11,623 (0.08%)	-31,045 (-0.40%)	-11,460 (-0.18%)
**Very high (0.90—1.00)**	98 (0.00%)	0 (0.0%0)	0 (0.00%)	0 (0.00%)	-98 (0.00%)	0 (0.0%0)	0 (0.00%)

### Future invasion risk

There was no significant difference between the areas of suitability between present, RCP 4.5, RCP 6.0 and RCP 8.5 scenarios (ANOVA: *F = 0*.*09*, *P = 0*.*97*, *df = 3*). However, there was an increase of 0.37% in the area with suitability category >0.5 under RCP 8.0, 0.79% under RCP 4.5 and 1.04% under RCP 6.0 (Fig 2 and Table 2). The currently invaded regions are even more prone for invasion in the future. High risk of invasibility under climate change was seen mainly in Daman and Diu (Union Territory), Bihar, Odisha, Lakshadweep Islands, Andaman and Nicobar Islands, parts of Karnataka, Jharkhand, Nagaland and Assam (Table 3). Expansion might occur in Jharkhand, Lakshadweep Islands, Andaman and Nicobar Islands, Odisha, Gujarat and Karnataka under RCP 4.5 scenario. Under RCP 6.0, Bihar, Andhra Pradesh, Gujarat, Andaman and Nicobar Islands, Odisha, Dadra and Nagar Haveli, Daman and Diu and Jharkhand were highly vulnerable. Under RCP 8.5, Gujarat, Jharkhand, Lakshadweep Islands, Andaman and Nicobar Islands, Bihar and Odisha have high risk of invasion. The risk of invasion is very low under all the three scenarios for the Central Indian region where there is high summer temperature and low rainfall and high altitude regions with cold climate in the Himalayas. It is interesting to note that the two states Tripura and Goa the invasion risk under all future climate scenarios is either very low or nil (Table 3). The percentage of land area with more than 0.5 invasion risk under the four scenarios for other Indian states is provided in S1 Table.

**Table 3:** Top 10 Indian states with >0.50% probability of invasion risk from *A*. *fulica* under present and future climate change scenarios. The values in table are in percentage.

Sl no	STATE	Present	RCP 4.5	RCP 6.0	RCP 8.5
1	West Bengal	87.93	78.65	75.83	76.08
2	Bihar	80.58	81.01	80.67	82.27
3	Kerala	69.23	63.76	59.56	39.97
4	Assam	51.57	46.43	54.84	55.72
5	Tripura	44.35	0.88	0.28	0
6	Jharkhand	41.71	57.83	50.85	55.05
7	Andaman and Nicobar Is	40.41	55.15	53.8	77.44
8	Goa	30.48	2.51	0	0
9	Karnataka	30.11	34.96	33.91	28.8
10	Lakshadweep Is	29.41	76.47	29.41	47.06

### Model performance and influencing factors

The MaxEnt model prediction has high AUC for both training and test data for all four scenarios (>0.94) (Table 4 and S1–S4 Figs) indicating good model performance for *A*. *fulica*. Annual Mean Temperature (Bio 1), Temperature Annual Range (Bio 7) and Annual Precipitation (Bio 12) are the three major environmental variables contributing to the risk of invasion by *A*. *fulica*, together accounting for nearly 30% to 45% under all four scenarios. Bio 4 (Temperature Seasonality) and Bio 15 (Precipitation Seasonality (Coefficient of Variation)) are also major contributors to invasion risk (Table 5).

**Table 4:** Area under curve for training and test points under different scenarios.

	No. of points	Present	RCP 4.5	RCP 6.0	RCP 8.5
**Training AUC±SD**	172	0.965±0.003	0.964±0.002	0.966±0.002	0.964±0.002
**Test AUC±SD**	59	0.953±0.011	0.953±0.005	0.949±0.007	0.953±0.008

**Table 5:** Relative contribution of different bioclimatic variables to MaxEnt model for *A*. *fulica*. Percent contribution values are averages over 10 replicate runs.

Variables	Current	RCP 45	RCP 60	RCP 85
Annual Mean Temperature (Bio1)	27.3	34.5	30.8	32.6
Temperature Seasonality (standard deviation *100 (Bio 4)	8.6	10.4	11.3	7
Temperature Annual Range (Bio7)	12.9	24.5	23.1	28.3
Annual Precipitation (Bio 12)	40	16	18.3	15
Precipitation of Driest Month (Bio14)	2	4.7	3.9	2.2
Precipitation Seasonality (Coefficient of Variation) (Bio 15)	7.9	8.2	10.4	13.8
Precipitation of Coldest Quarter (Bio19)	1.3	1.7	2.2	1.1

**Fig 3:**
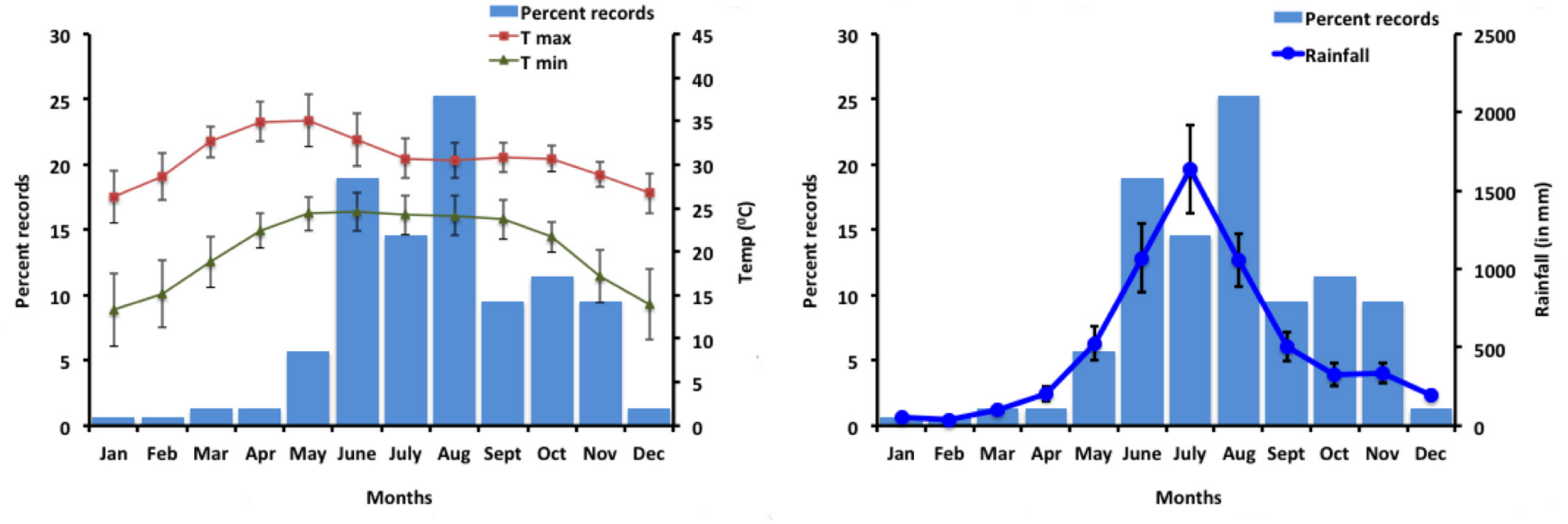
a) Occurrence of *A*.*fulica* in different months of the year (n = 158) with min and max temperature; b) Occurrence of *A*.*fulica* in different months of the year (n = 158) with average rainfall.

**Fig 4:**
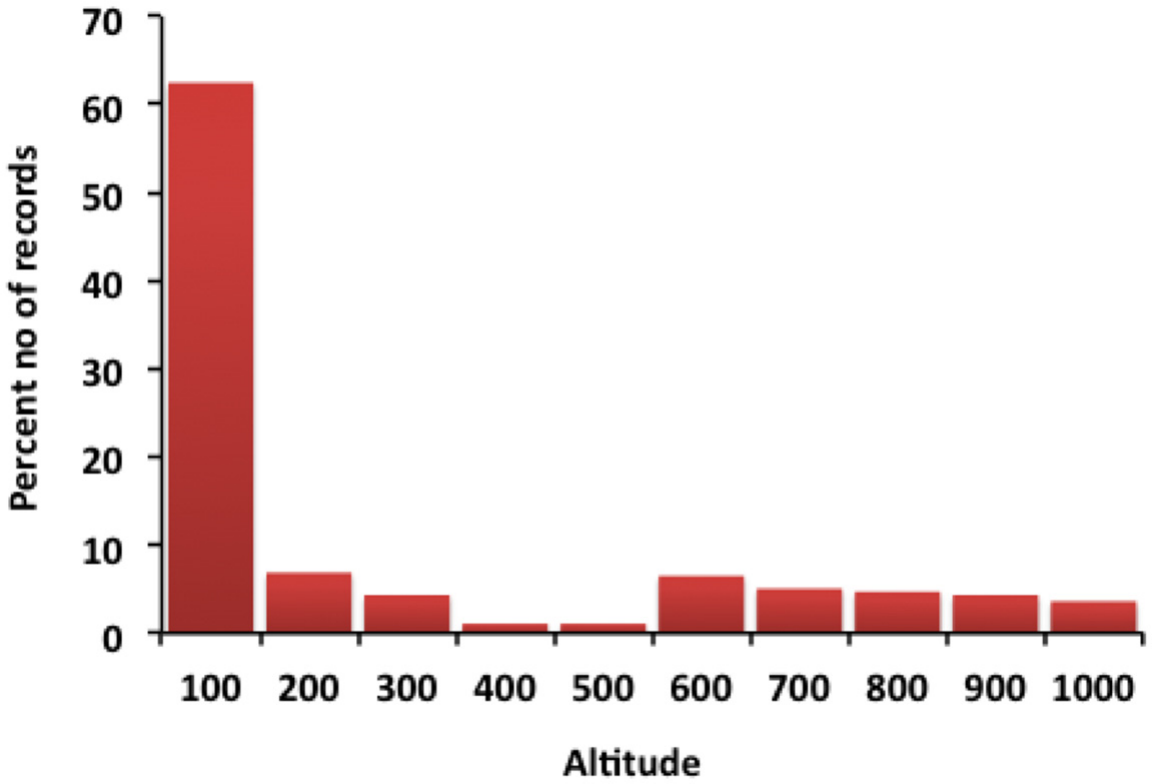
Percent number of records of *A*.*fulica* invasion along the altitudinal gradient.

Among different climatic factors, average minimum monthly temperature (°C) (*r = 0*.*672*, *P<0*.*05*, *df = 10;* Fig 3a) and average monthly rainfall (mm) (*r = 0*.*820*, *P<0*.*01*, *df = 10;* Fig 3b) are significantly correlated with occurrence of *A*. *fulica* in India (Table 6). Most records of *A*. *fulica* were from altitudes ranging from 1-100m asl (62.45%; Fig 4). There were few records from above 1000m asl.

**Table 6:** Pearson’s correlation between number of *A*. *fulica* records and climatic factors.

	No. of records	Temp max	Temp min	Rainfall
**No. of records**	1.000			
**Temp max (°C)**	0.161			
**Temp min (°C)**	**0.672** *	**0.771** **		
**Rainfall (mm)**	**0.820** **	0.232	**0.701** *	1.000

*P<0.05,

**P<0.01,

df = 10

## Discussion

The impact of future climate change on distribution in a wide range of species has been predicted [62], [63], but the magnitude of the potential impact on biodiversity remains largely unknown [64]. Not all organisms respond in the same way across different spatial and temporal scales. Hence, specific information on a species’ response is required for effective conservation and management of biodiversity. Species may respond to climate change by adapting to changing environmental conditions, shifting their niche, expanding to newer areas or in the worst case scenario become extinct if unable to adapt to changing environment [65]. Niche models can be helpful in predicting species’ distribution in relation to climate change. The climate change and invasive species together can have a drastic effect not only on biodiversity [15] but also on human well-being [66,67]. ENM has been used extensively to predict future ecologically suitable areas for the establishment of invasive alien species under changed climate scenario [17,68–70].

### Current and future distribution

The maximum entropy niche modeling procedure was adopted here to understand *A*. *fulica* distributions in India under present conditions and three climate change scenarios and to identify the areas most vulnerable to invasion under changed climate. The models show high AUC values (>0.94 for both test and training data) indicating that models successfully predict species distribution with a high degree of accuracy. An earlier work by Sridhar et al. [71] using CLIMEX model to predict *A*. *fulica* invasion under current climate scenario used much coarser grid (0.5° degrees) than the present study (30 arc seconds). This study showed that much of Himachal Pradesh, Uttarakhand, Jammu and Kashmir, the warmer and drier regions of Rajasthan and Gujarat and some parts of North Eastern states are less vulnerable to invasion, which is also shown in our study. Apart from these states, most parts of India are highly prone to invasion [69]. However, contrary to this, our study being at much finer scale emphasizes that not all parts of each state are equally vulnerable to invasion. Another study at a much smaller scale using maximum entropy modeling has predicted an increased infestation of *A*. *fulica* in Kerala [72]. However, our results suggest that there is no significant difference between invasion risks in Kerala under current and all three future climate change scenarios.

Not all invasive species respond to climate change in a similar way. For example, some invasive species are likely to be reduced in range due to climate change [11,73,74]. The studies have suggested that American bullfrog and Burmese python will have reduced invasion potential under climate change scenarios [74,75]. A recent study on Argentine ant invasions at global level have predicted reduced invasive potential, but there is potential for some regional expansions [76]. On the contrary, increase in temperature expands range for some other invasive species like *Chromolaena odorata* and *Lantana camara* [51,77]. In the Indian scenario, studies speculate that change in climate will increase wetness and precipitation [78], especially in the southern Western Ghats and some species will undergo shifts in their range in future [79,80]. The MaxEnt model results suggests that the climate change (under RCP 6.0) is likely to increase land area at risk of invasion by up to 1.04%. Currently less suitable regions might become more suitable and vice-versa under changed climate, especially in peninsular India, and eastern India.

### Economic and conservation impacts

The economic costs of agriculture associated with invasions are significant due to loss of productivity and resources spent on control efforts [14]. Naylor [81] estimated that the total cost of *Pomacea canaliculata* (apple snail) invasion to Philippine rice agriculture in 1990 was US$ 425–1200 million. This estimate does not include impacts on ecosystems and human health. The economic loss due to *Dreissena polymorpha* (zebra mussel) invasion in the United States was estimated to be US$ 3.1 billion between 1993–1999 for power plants and over US$ 5 billion when other sectors are considered [82] and in Canada, impact was estimated at US$ 376,000 annually per generating station estimated in 1994 [82]. In the United States, management authorities took nearly a decade and US$ 1 million to completely eradicate *A*. *fulica* from Florida [83]; however, there are several recent reports of *A*. *fulica* occurrence in this region despite all the efforts.

The invasion of *A*. *fulica* in India is a major problem, not only in semi-urban or peri-urban areas but also increasingly in rural areas. Agriculture, particularly in India with nearly 60% being rain-fed, has been a highly risky venture with extremities in monsoon coupled with other abiotic and biotic factors. Climate change is set to compound the daunting complex challenges already being faced by the farmers. Increasing temperature will have significant negative impact on crop yield [84]. This along with land use and invasive species such as *A*. *fulica* will have even more impact on small-scale agriculture and horticulture. The invasion is particularly high during monsoon months (June to November) when most of the small-scale farmers grow vegetables and horticultural crops. We have found here that regions with high agriculture output in India also have high invasion risk. These regions include Kerala, coastal parts of Karnataka and Andhra Pradesh, Tamil Nadu, Maharashtra and Assam [85]. The slow growth regions in terms of agriculture such as Bihar and parts of Uttar Pradesh are also very economically backward regions and highly dependent on monsoon for good yield. These regions are also highly vulnerable to invasion. Increased invasion by *A*. *fulica* under future climate in these regions will only worsen the agriculture situation in India, thus further marginalizing the small-farming community and adversely impacting local economies. *A*. *fulica* not yet invaded forested regions of India. The impact of snail invasion on native snail fauna is not known. Given the high endemism in land snails in India [86,87], there is urgent need to study the impact on native biodiversity.

### Factors influencing invasion

The results of niche modelling of *A*. *fulica* in India show that most of the invasion risk is in the regions with high rainfall and warm climate. The regions with extreme cold and heat are less vulnerable to invasion under current as well as in future climate scenario. *A*. *fulica* have a broad altitudinal (1-1000m asl), precipitation (350-5000m/year) and temperature range (0°C to 45°C), but with an optimal temperature range from 22°C to 32°C [25]. The ideal condition for their growth and activity is a good amount of rainfall, humidity, shade and optimum temperature [80,88]. In our study also, in all three scenarios, temperature is the major influencing factor for distribution of *A*. *fulica*. Our model has predicted annual mean temperature, temperature seasonality and temperature annual range to be the major factors determining the invasion in all scenarios reinforcing the above results [88]. The highest infestation takes place with the onset of monsoon (June) and remains active throughout the rainy season and start declining gradually from mid-November [89,90]. Our study shows that the maximum invasion risk occurs between June and November during which India receives monsoon [91] (Fig 3b). Apart from temperature and rainfall, altitude also plays a significant role in *A*. *fulica* invasion. Raut and Ghose [25] reported that hilly regions of eastern and northern India above 1500m were not suitable due to low temperature. Raut [92] reported that population of *A*. *fulica* decreased with the increase in altitude, this is also seen in our study. Thus, *A*. *fulica* distributions seem to be predominantly influenced by altitude, mean temperature and precipitation.

### Management and policy requirements

The development of management policies is the key to controlling the spread of *A*. *fulica*. These policies should be based on sound science that takes into account interactions between the species, climate change, land use changes and livelihood aspects of the communities in the invaded landscape [93]. Since, the snail is a major pest on agricultural and horticultural crops, it is imperative to consider livelihood while framing the policies. In particular, small farmers are highly vulnerable to both invasive species as well as climate change. Hence, the cost of invasion on agriculture and horticulture needs to be studied.

Ecological niche modeling is a cost-effective, easy and early warning system that allows the identification of areas at risk from a potential invasion, thus giving the opportunity to prioritize the region and target management actions as well as investment of resources in those certain regions. There is an urgency to set up long-term monitoring studies on *A*. *fulica* populations to understand the invasion patterns, its impact on native species and economy that will allow better understanding for future management of this highly invasive land snail. In order to control the spread of the existing *A*. *fulica* populations, and to prevent further invasions in India, we consider that the results of this study should be taken into account when identifying vulnerable areas and making management decisions for control of *A*. *fulica* in India.

## Supporting Information

S1 Fig Jackknife test of variable importance for *A*.*fulica* under present scenario. (PDF)

S2 Fig Jackknife test of variable importance for *A*.*fulica* under RCP 4.5 scenario. (PDF)

S3 Fig Jackknife test of variable importance for *A*.*fulica* under RCP 6.0 scenario. (PDF)

S4 Fig Jackknife test of variable importance for *A*.*fulica* under RCP 8.5 scenario. (PDF)

S1 Table List of all Indian States with > 0.5 probability of invasion risk under different climate change scenarios. The values are in percentage
(PDF)
