# Supplementary figures and images for: Effect of Climate Change on Invasion Risk of Giant African Snail (Achatina fulica Férussac, 1821: Achatinidae) in India
Source: PLoS One. 2015 Nov 30;10(11):e0143724. doi: 10.1371/journal.pone.0143724 (PMC4664396; doi:10.1371/journal.pone.0143724)

**S1 Fig: Jackknife test of variable importance for *A.fulica* under present scenario**

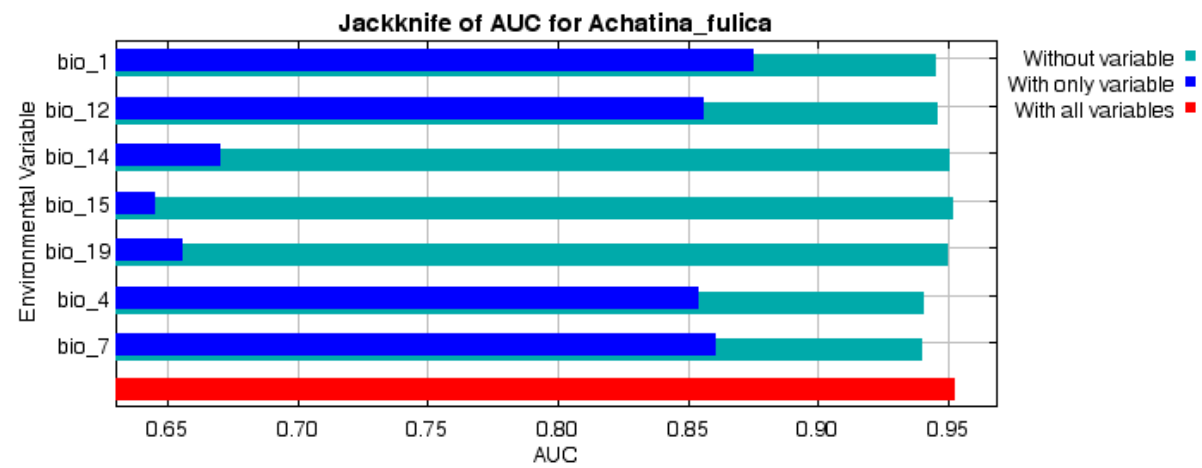

Supplement: S1 Fig — (PDF) [file pone.0143724.s001.pdf]

**S2 Fig: Jackknife test of variable importance for *A.fulica* under RCP 4.5 scenario**

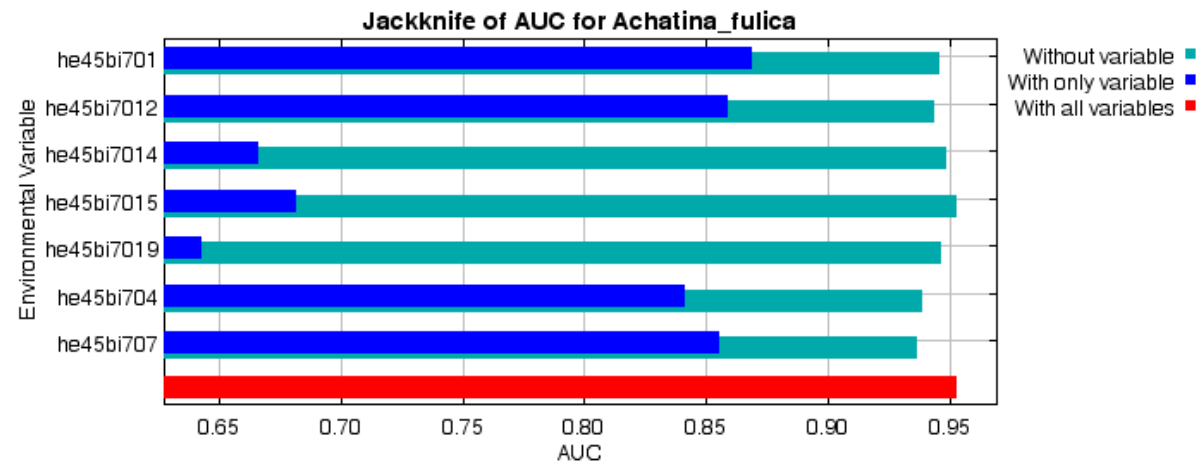

Supplement: S2 Fig — (PDF) [file pone.0143724.s002.pdf]

**S3 Fig: Jackknife test of variable importance for *A.fulica* under RCP 6.0 scenario**

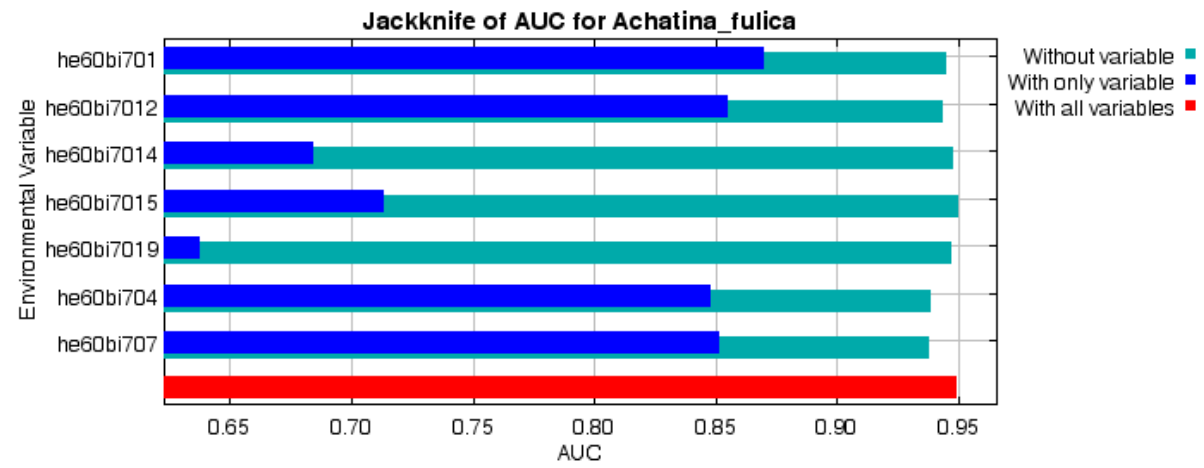

Supplement: S3 Fig — (PDF) [file pone.0143724.s003.pdf]

**S4 Fig: Jackknife test of variable importance for *A.fulica* under RCP 8.5 scenario**

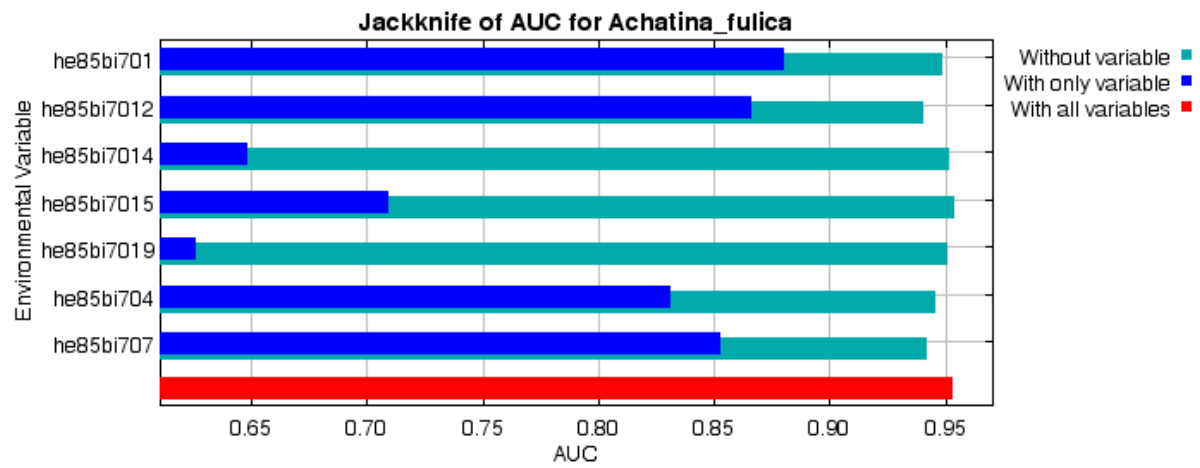

Supplement: S4 Fig — (PDF) [file pone.0143724.s004.pdf]
