# Supplementary material for: Effect of Climate Change on Invasion Risk of Giant African Snail (Achatina fulica Férussac, 1821: Achatinidae) in India
Source: PLoS One. 2015 Nov 30;10(11):e0143724. doi: 10.1371/journal.pone.0143724 (PMC4664396; doi:10.1371/journal.pone.0143724)
Supplement: S1 Table — The values are in percentage (PDF) [file pone.0143724.s005.pdf]

S1 Table: List of all Indian States with > 0.5 probability of invasion risk under different climate change scenarios. The values are in percentage

STATE	present	RCP 4.5	RCP 6.0	RCP 8.5
WEST BENGAL	87.93	78.65	75.83	76.08
BIHAR	80.58	81.01	80.67	82.27
KERALA	69.23	63.76	59.56	39.97
ASSAM	51.57	46.43	54.84	55.72
TRIPURA	44.35	0.88	0.28	0.00
JHARKHAND	41.71	57.83	50.85	55.05
ANDAMAN NICKOBAR	40.41	55.15	53.80	77.44
GOA	30.48	2.51	0.00	0.00
KARNATAKA	30.11	34.96	33.91	28.80
LAKSHADWEEP	29.41	76.47	29.41	47.06
MEGHALAYA	22.15	6.71	0.84	6.03
ORISSA	8.40	13.59	12.86	16.23
TAMILNADU	7.97	4.35	3.11	3.61
MIZORAM	5.07	2.08	5.64	2.82
UTTAR PRADESH	3.06	2.26	1.52	2.41
MAHARASHTRA	2.95	1.64	3.01	1.74
PONDICHERRY	1.70	1.68	1.68	1.68
MANIPUR	1.46	0.00	0.27	0.01
ANDHRA PRADESH	1.46	3.77	3.89	5.47
SIKKIM	0.51	0.00	0.01	0.00
NAGALAND	0.48	3.09	7.11	4.78
ARUNACHAL PRADESH	0.44	0.30	0.58	0.25
CHANDIGARH	0.00	0.00	0.00	0.00
CHHATTISGARH	0.00	0.00	0.00	0.02
DADRA AND NAGAR HAVELI	0.00	0.00	10.16	0.00
DAMAN AND DIU	0.00	0.00	21.28	0.00
DELHI	0.00	0.00	0.00	0.00
GUJARAT	0.00	2.01	2.14	2.03
HARYANA	0.00	0.00	0.00	0.00
HIMACHAL PRADESH	0.00	0.00	0.00	0.07
JAMMU & KASHMIR	0.00	0.00	0.00	0.00
MADHYA PRADESH	0.00	0.00	0.00	0.00
PUNJAB	0.00	0.00	0.00	0.00
RAJASTHAN	0.00	0.00	0.03	0.00
UTTARAKHAND	0.00	0.06	0.00	0.06
